# The quality of reporting in case reports of permanent neonatal diabetes mellitus: a cross-sectional study

**DOI:** 10.1186/s12874-024-02226-1

**Published:** 2024-05-20

**Authors:** Pengli Jia, Ling Wang, Xi Yang, WenTing Pei, Chang Xu, Jinglin Feng, Ying Han

**Affiliations:** 1 School of Management, Shanxi Medical University, Taiyuan, China; 2 The Second Clinical Medical College of Anhui Medical University, Hefei, China; 3 Department of Maternal, Child and Adolescent Health, School of Public Health, Anhui Medical University, Hefei, China; 4 Proof of Concept Center, Eastern Hepatobiliary Surgery Hospital, Third Affiliated Hospital, Second Military Medical University, Naval Medical University, Shanghai, China

**Keywords:** Permanent neonatal diabetes mellitus, Case report, CARE, Reporting quality

## Abstract

**Background:**

Although randomized trials and systematic reviews provide the best evidence to guide medical practice, many permanent neonatal diabetes mellitus (PNDM) studies have been published as case reports. However, the quality of these studies has not been assessed. The purpose of this study was to assess the extent to which the current case reports for PNDM comply with the Case Report (CARE) guidelines and to explore variables associated with the reporting.

**Method:**

Six English and four Chinese databases were searched from their inception to December 2022 for PNDM case reports. The 23 items CARE checklist was used to measure reporting quality. Primary outcome was the adherence rate of each CARE item and second outcome was total reporting score for each included PNDM case report. Linear and logistic regression analyses were used to examine the connection between five pre-specified predictor variables and the reporting quality. The predictor variables were impact factor of the published journal (<3.4 vs. ≥3.4, categorized according to the median), funding (yes vs. no), language (English vs. other language), published journal type (general vs. special) and year of publication (>2013 vs. ≤ 2013).

**Result:**

In total, 105 PNDM case reports were included in this study. None of the 105 PNDM case reports fulfilled all 23 items of the CARE checklist. The response rate of 11 items were under 50%, including prognostic characteristics presentation (0%), patient perspective interpretation (0%), diagnostic challenges statement (2.9%), clinical course summary (21.0%), diagnostic reasoning statement (22.9%), title identification (24.8%), case presentation (33.3%), disease history description (34.3%), strengths and limitations explanation (41.0%), informed consent statement (45.7%), and lesson elucidation (47.6%). This study identified that the PNDM case reports published in higher impact factor journals were statistically associated with a higher reporting quality.

**Conclusion:**

The reporting of case reports for PNDM is generally poor. As a result, this information may be misleading to providers, and the clinical applications may be detrimental to patient care. To improve reporting quality, journals should encourage strict adherence to the CARE guidelines.

**Supplementary Information:**

The online version contains supplementary material available at 10.1186/s12874-024-02226-1.

## Background

Neonatal diabetes mellitus (NDM) is a rare metabolic disease with an incidence of 90,000-160,000 neonates [1]. The permanent form of neonatal diabetes mellitus (PNDM) accounts for approximately half of all cases, with an incidence of one in 260,000 live births [2]. PNDM is a lifelong disease without remission that requires treatment throughout life [3]. The main clinical manifestations are hyperglycemia, intrauterine growth retardation, ketoacidosis, weight loss and reduced quality of life [4]. Given the severe condition and substantial medical need of PNDM, there is an urgent need for high-quality clinical research to guide PNDM clinical practice [5].

However, traditional clinical research methods for PNDM are often impeded by the scarcity and geographical dispersion of patients and the involvement of children, which can result in deficiencies in the development of clinical research evidence [6]. For example, Tudur found that compared to non-rare disease clinical trials, rare disease clinical trials are single-arm, non-randomized, non-blind, open-label, and too fragile to be terminated early [7]. Given the problems with recruitment in PNDM research, innovative strategies for rare disease clinical research are urgently required for high-quality diagnosis and treatment evidence [5].

Case reports have been used to recognize the genetic cause, main symptoms, medical, family, or psychosocial history, and clinical diagnostic, therapeutic, and prognostic information of PNDM [8–11]. However, there is a continuing debate about the validity of PNDM case reports and their value to practicing clinicians [12]. These case reports are generally regarded as having poor evidential quality because of their prose and spontaneous reporting [13]. Written without the benefit of reporting guidelines, case reports are often insufficiently rigorous to be aggregated for data analysis, to inform research design, or to guide clinical practice [13].

Surprisingly, general international reporting guidelines for case reports did not exist until the CARE (CAse REport) Guidelines were published [13]. Although PNDM case reports are overrepresented in the literature, little is known about reporting quality. A lack of adequate reporting of details would make the effective use of such case reports evidence less likely. Under certain circumstances, this can lead misinformed healthcare decisions. Therefore, this study conducted a cross-sectional study to specifically assess the extent to which the current case reports for PNDM complied with the CARE guidelines and explore factors associated with reporting.

## Methods

### Inclusion criteria

All case reports enrolled patient diagnosed with PNDM will be included. PNDM was defined as a diagnosis of diabetes within 4 or 6 weeks of birth [3]. An included case report should report useful clinical information on PNDM, such as clinical findings, patient characteristics, diagnosis or therapeutic information. There was no limitation on the publication language.

### Literature search and screening

This study searched PubMed, EMBASE, Scopus, Web of Science, CINAHL, Medrxiv, and four Chinese Databases, SinoMed, National Knowledge Infrastructure (CNKI), Wanfang, and VIP, from inception to 1st of December 2022. A combination of keywords and Medical Subject Headings related to PNDM and case report was used ("pediatric”, “PNDM”, “NDM”, "permanent neonatal diabetes mellitus”, "case report”, "WRS” and "Wolcott-Rallison syndrome"). The reference lists of eligible papers were also manually screened for articles that were not identified by the computerized search. Further details are provided in Appendix 1.

Pairs of well-trained authors, independently and in duplicate, scanned titles and abstracts to exclude obviously irrelevant studies, and potentially eligible articles were investigated in full text. Disagreements were resolved by discussion between the two reviewers; if no consensus was achieved, a third reviewer was involved.

### Data collection

Data extraction was performed by two authors using a predefined data sheet that included general publication information: name of the first author, year of publication, published language, region of the first author, funding information, journal where the care report was published, and the journal’s impact factor.

The CARE guidelines checklist was used to assess the reporting quality of case reports [15]. We slightly modified the checklist by merging some sub-items into one item: 1) the four sub-item “the main symptoms of the patient, main clinical findings, the main diagnoses and interventions and the main outcomes” were merged as item 3b “Case Presentation”; 2) types of intervention (eg, pharmacologic, surgical, preventive, self-care), administration of intervention (eg, dosage, strength, duration) and changes in intervention (with rationale) were merged as item 9 “therapeutic intervention”; 3) clinician and patient-assessed outcomes, important follow-up test results (positive or negative), intervention adherence and tolerability (and how this was assessed) and adverse and unanticipated events were merged as item 10 “clinical course of all follow-up visits”; The merging resulting in 23 items of the finally CARE guideline checklist, see details in the Appendix 2.

### Outcome

For each included PNDM case report, quality of reporting against the 23 items was determined as “Yes”, “Partially yes”, or “No”. The primary outcome was Adherence Rate. The Adherence rate (AR=n/N) and 95% confidence interval (CI) were used to reflect the degree of compliance of each case report to each item of CARE checklist, where n is the number of PNMD case reports adhering to the requirement of a certain item, and N is the total number of PNMD case reports. The present study summarized the AR of each item at three levels: met by 80% or above was well complied, 50 to 79% was moderately complied, and less than 50% was poorly complied.

The second outcome was the total score of reporting. The item rated as “Yes” “Partially yes” or “No” was given a point of 2, 1 or 0 respectively. Possible scores ranged from 0 to 46. Higher scores indicated better quality. The purpose of the score was to explore the connections between some pre-specified factor and reporting quality.

### Data analysis

Baseline characteristics which included multinomial (language, region of first author, impact factor of the published journal) and dichotomous variables (year of publication, published journal type, sources of funding) were described as number and percentages.

This study pre-specified five variables to explore their connection to reporting quality. These were impact factor of the published journal (<3.4 vs. ≥3.4, categorized according to the median), funding (yes vs. no), language (English vs. other language), journal type (general vs. special) and year of publication (≤ 2013 vs. >2013). The year was categorized based on the year CARE was published. Reporting scores of the five pre-specified group were calculated as median and interquartile ranges (IQR). Standardized β coefficient with 95% confidence intervals (CI) were calculated using univariate and multivariate linear regression analyses to examine the association between reporting score and the pre-specified variables.

In order to avoid the bias of the score system on the results, we conducted a logistic regression in which the adherence to each 23 items CARE checklist was categorized as two group (Yes or No), the predictor factor was “published journal (<3.4 vs. ≥3.4, categorized according to the median), funding (yes vs. no), language (English vs. other language), published journal type (general vs. special) and year of publication (≤ 2013 vs. >2013). Standardized Odds Ratio (OR) with 95% CI was estimated by the logistic regression to examine the association between response quality and the five variables.

All the analyses were conducted using Stata14.0/SE software (STATA, College Station, TX, Serial number: 10699393), and alpha = 0.05 was the criterion for statistical significance.

## Results

**Fig. 1:**
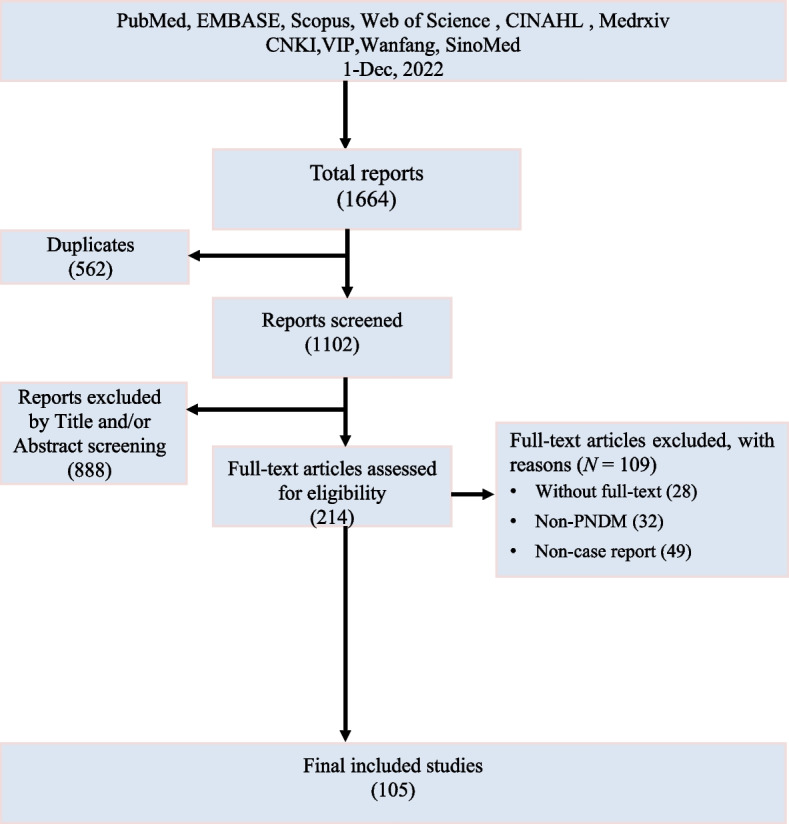
Flow plot of literature search and screening

The initial search yielded 1664 reports, of which 1316 were eliminated due to duplication or title and abstract screening. After full-text reading, 105 case reports on PNDM were included. No additional case reports were identified through the reference list screening (Fig. 1).

### Characteristics of included studies

A total of 105 PNDM case reports were published between 1971 and December 2022. The majority were published in English (93.33%). Research groups from Asian contributed most (40.00%), followed by European (38.09%), and North American (17.14%) groups. Majority of case reports were published in specialized journals (86.67%), such as pediatrics and endocrinology. The median impact factor for the published journals was 3.40 (IQR: 1.48, 4.50). Almost half of the included cases reported funding resources (57/105), all of which were provided by nonprofit funding agencies (Table 1).

**Table 1:** General characteristics of included case reports

**Features of included case reports**	**Total (*n*=105, %)**
**Year of publication**	
≤ 2013	54 (51.4)
>2013	51 (48.6)
**Language**	
English	98 (91.6)
Chinese	5 (4.8)
Polish	1 (0.1)
Croatian	1 (0.1)
**Region of first author**	
Asian	42 (43.9)
European	40 (37.4)
North American	18 (16.8)
African	3 (2.8)
South American	2 (1.9)
**Type of journals**	
Specialist journal	91 (86.7)
General journal	14 (13.3)
**IF of the published journals**	
<3.4	42 (40.0)
≥3.4	46 (43.8)
None	17 (16.2)
**Sources of funding**	
Non-profit funding agencies	57 (54.3)
No funding or funding not reported	48 (45.7)

*IF* Impact factor

### Adherence rate of each reporting item

The overall CARE scores resulted in a median score of 28 (IQR: 23, 30). None of the 105 PNDM case reports fulfilled all 23 items of the CARE checklist: five out of 23 items were well complied, seven were moderately complied, and 11 were poorly complied. The adherence rates for the items reported in the CARE checklist are listed in Table 2.

**Table 2:** The Adherence rate (AR) of reporting quality by CARE guidelines checklist

**Items**	**Brief description**	**Yes** **n (%)**	**Partial** **n (%)**	**No** **n (%)**
Title	1. Case report/study in title	26 (24.8)	79 (75.2)	0 (0.0)
Keywords	2. 2-5 words	65 (61.9)	8 (7.6)	32 (30.5)
Abstract	3a. Introduction	63 (60.0)	2 (1.9)	40 (38.1)
	3b. Case Presentation^a^	35 (33.3)	42 (40.0)	28 (26.7)
	3c. Conclusion	50 (47.6)	1 (1.0)	54 (51.4)
Introduction	4. Brief background summary	83 (79.0)	2 (1.9)	20 (19.0)
Patient information	5a. Demographic information	101 (96.2)	4 (3.8)	0 (0.0)
	5b. Main symptoms of the patient	59 (56.2)	1 (1.0)	45 (42.9)
	5c. Medical, family, and psychosocial history	36 (34.3)	62 (61.0)	5 (4.8)
Clinical findings	6. Physical examination findings	94 (89.5)	3 (2.9)	8 (7.6)
Timeline	7. Important dates and times	59 (56.2)	2 (1.9)	44 (41.9)
Diagnostic assessment	8a. Diagnostic methods	99 (94.3)	0 (0.0)	6 (5.7)
	8b. Diagnostic challenges	3 (2.9)	0 (0.0)	102 (97.1)
	8c. Diagnostic reasoning	24 (22.9)	42 (40.0)	39 (37.1)
	8d. Prognostic characteristics	0 (0.0)	0 (0.0)	0 (0.0)
Therapeutic intervention	9. Types of intervention^a^	79 (75.2)	19 (18.1)	7 (6.7)
Follow-up and outcomes	10. Clinical course of all follow-up^a^	22 (21.0)	57 (54.3)	26 (24.8)
Discussion	11a. Strengths and limitations	43 (41.0)	19 (18.1)	43 (41.0)
	11b. Relevant medical literature	90 (85.7)	1 (1.0)	14 (13.3)
	11c. Rationale for conclusions	89 (84.8)	1 (1.0)	15 (14.3)
	11d. Main ‘take-away’ lessons	69 (65.7)	1 (1.0)	35 (33.3)
Patient perspective	12. Patient perspective or experience whenever possible	0 (0.0)	0 (0.0)	105 (100)
Informed consent	13. Informed consent	48(45.7)	0 (0.0)	57 (54.3)

The details of 23 items CARE guidelines checklist were showed in Appendix 2

^a^The revised items by merging sub-items

The title section item, which was identified as “elucidated the study as ‘case report’ along with phenomenon of greatest interest”, was poorly complied (AR=24.8%, 95% CI: 16.4, 33.2%). The keyword element describing the key information of the case as 2-5 words was moderately complied with 61.9% (95% CI: 52.5, 71.3%) of the PNDM case reports adhering this item.

Of the three items in the abstract section, the item of introduction narration was moderately complied (AR=60.0%, 95% CI:50.5, 69.5%), while the other two items were poorly complied: case presentation (AR=33.3%, 95% CI:24.2, 42.5%) and lesson elucidation (AR=47.6%, 95% CI:37.9, 57.3%). The background summary was complied by 79.0% (95% CI: 71.1, 87.0%) of the PNDM case repots.

In terms of the patient information (three items), 59 (AR=56.2%, 95 CI:46.5, 65.8%) provided details of demographic information, and a large proportion (AR=96.2%, 95% CI: 92.5, 99.9%) specified the main symptoms of the patient, while only a small proportion (AR=34.3%, 95% CI:25.1, 43.5%) specified details regarding the medical, family, and psychosocial history.

Within the diagnostic assessment element, there were 4 items identified, including clarifying the diagnostic methods (AR=94.3%, 95% CI: 89.8, 98.8%), diagnostic reasoning (AR=22.9%, 95% CI: 14.7, 31.0%.), diagnostic challenges (AR=2.9%, 95% CI: -0.4, 6.1%) and prognostic characteristics (AR=0%).

Of the four items in the discussion section, relevant medical literature, rationale for conclusion and main take-away’ lessons were evaluated completely in 90 (AR=85.7%, 95% CI: 78.9, 92.5%), 89 (AR=84.8%, 95% CI: 77.8, 91.8%) and 69 (AR=65.7%, 95% CI: 56.5, 74.9%) PNDM case reports, respectively. Total compliance was less than 50% in the strengths and limitations item (41.0%, 95%CI: 31.4, 50.5%).

With regard to the four separately specified items, description of physical examination (AR=89.5%, 95%CI:83.6, 95.5%) was highly adhered, types of intervention (AR=75.2%, 95%CI:66.8, 83.6%) and important dates and times (AR=56.2%, 95%CI:46.5, 65.8%) were moderately adhered. The remaining item summarized the clinical course of all follow-up visits (AR=21.0%, 95%CI:13.0, 28.9%) was poorly addressed.

For the two alternative items, informed consent was poorly complied (AR=45.7%, 95CI: 36.0, 55.4), while the reporting of patient perspective was seriously limited (AR=0%).

### Factors associated with the reporting quality

The median and IQR of reporting score in the case reports published with funding, in English language and after year 2013 were 27.0 (23.5 to 30.5), 27.5 (23.7, 30.0) and 28.0 (24.0, 31.0). For those case reports that in general and impact factor ≥3.4 journals, the median and IQR of reporting score were 25.0 (21.2, 29.0) and 27.0 (22.0, 29.0).

Multivariable linear regression analyses showed that PNDM case reports published in higher impact factor journals were statistically associated with a higher total score (standardized β coefficient 0.27, 95% CI: -4.98 to 0.59), while those published in recent years (standardized β coefficient 0.12, 95% CI: -0.89 to 3.46), in English (standardized β coefficient -0.14, 95% CI: -7.08 to 1.48), in a general journal (standardized β coefficient -0.17, 95% CI: -5.79 to 0.50), and with funding supporting (standardized β coefficient -0.90, 95% CI: -3.09 to 1.29) were not associated with the reporting (Table 3).

**Table 3:** The multivariable linear regression results of factors associated with overall reporting quality

**Variables**	**Coefficient** **(95% CI)**^**a**^	***t***	***P***	**Coefficient** **(95% CI)**^**b**^	***t***	***P***
**Language**						
English vs. others_[Ref]_	-0.06 (-5.24 to 2.83)	-0.59	0.56	-0.14 (-7.08 to 1.48)	-1.30	0.19
27.5 (23.7, 30.0) vs. 31.0 (22.0, 34.0)^c^						
**Publication year**						
>2013 vs. ≤ 2013_[Ref]_	0.18 (-0.18 to 3.79)	1.80	0.08	0.12 (-0.89 to 3.46)	1.18	0.24
28.0 (24.0, 31.0) vs. 27.0 (22.0, 29.0)^c^						
**Impact factor of published journals**						
≥3.4 vs. <3.4_[Ref]_	**0.26 (-4.93 to 0.59)**	**-2.52**	**0.01**	**0.27 (-4.98 to 0.59)**	**-2.52**	**0.01**
27.0 (22.0, 29.0) vs. 29.0 (25.7, 32.2)^c^						
**Published journal type**						
General vs. special_[Ref]_	-0.14 (-5.02 to 0.86)	-1.41	0.16	-0.17 (-5.79 to 0.50)	-1.67	0.10
25.0 (21.2, 29.0) vs. 28.0 (24.0, 31.0)^c^						
**Funding**						
Yes vs. no_[Ref]_	-0.02 (-2.21 to 1.83)	-0.19	0.85	-0.09 (-3.09 to 1.29)	-0.82	0.42
27.0 (23.5 to 30.5) vs. 28.0 (23.0 to 30.0)^c^						

Coefficient was Standardized β coefficient

The R^2^ of the multivariable linear regression model was 0.36

Others: including Chinese, Polish and Croatian

_*[Ref]*_ Reference level

^a^Univariable linear regression analyses

^b^Multivariable linear regression analyses

^c^Median and interquartile ranges (IQR)

The multiple logistic regression showed that PNDM case reports published in English (OR 15.94, 95% CI 1.59, 160.16) and higher impact factor journals (impact factor ≥3.4) (OR 2.77, 95% CI 1.03, 7.40) were associated with a higher likelihood of case presentation. Similarly, PNDM case reports published in the higher impact factor journals were more likely to achieve reporting the conclusion (OR 3.21, 95% CI 1.29, 8.00) and brief background summary (OR 6.23, 95% CI 1.50, 25.71). PNDM case reports published in general journals (OR 7.53, 95% CI 1.43, 39.76) and with funding support (OR 3.78, 95% CI 1.45, 9.85) were associated with a higher likelihood of achieving informed consent (Table 4).

**Table 4:** The logistic regression results of factors associated with CARE guidelines checklist

**Items**	**OR(95%CI)**			
	**Crude**	***P***	**Adjusted**^**&**^	***P***^***&***^
**1. Case report/study in title**				
English vs. others^a^_[Ref]_	0.49 (0.06, 4.24)	0.51	0.61 (0.06, 5.75)	0.66
Publication year >2013 vs. ≤ 2013_[Ref]_	0.76 (0.31, 1.84)	0.54	0.93 (0.34, 2.54)	0.89
Impact factor of journals ≥3.4 vs. <3.4_[Ref]_	0.99 (0.37, 2.65)	0.99	0.99 (0.36, 2.74)	0.99
Published in general vs. special journal_[Ref]_	2.15 (0.45 10.31)	0.34	4.21 (0.50, 35.27)	0.19
Funding vs. no_[Ref]_	1.54 (0.63, 3.76)	0.34	1.48 (0.54, 4.07)	0.45
**2. 2-5 keywords**				
English vs. others^a^_[Ref]_	0.22 (0.41, 1.21)	0.08	0.27 (0.04, 1.77)	0.17
Publication year >2013 vs. ≤ 2013_[Ref]_	0.41 (0.18, 0.92)	0.03	0.61 (0.24, 1.55)	0.30
Impact factor of journals ≥3.4 vs. <3.4_[Ref]_	2.37 (0.96, 5.82)	0.61	2.01 (0.79, 5.15)	0.14
Published in general vs. special journal_[Ref]_	0.89 (0.28, 2.87)	0.84	0.71 (0.18, 2.80)	0.63
Funding vs. no_[Ref]_	0.46 (0.21, 1.03)	0.06	0.46 (0.18, 1.17)	0.10
**3a. Introduction**				
English vs. others^a^_[Ref]_	0.88 (0.19, 4.16)	0.87	1.20 (0.2, 7.13)	0.84
Publication year >2013 vs. ≤ 2013_[Ref]_	1.29 (0.59, 2.82)	0.52	1.34 (0.55, 3.26)	0.52
Impact factor of journals ≥3.4 vs. <3.4_[Ref]_	0.95 (0.40, 2.26)	0.91	0.91 (0.37, 2.25)	0.84
Published in general vs. special journal_[Ref]_	2.24 (0.72, 6.99)	0.17	2.62 (0.75, 9.17)	0.13
Funding vs. no_[Ref]_	0.75 (0.34, 1.64)	0.47	0.76 (0.31, 1.85)	0.54
**3b. Case Presentation**^**b**^				
English vs. others^a^_[Ref]_	5.67 (1.04, 30.87)	0.05	15.94 (1.59,160.16)	**0.02**
Publication year >2013 vs. ≤ 2013_[Ref]_	0.84 (0.37, 1.9)	0.68	0.71 (0.28, 1.85)	0.49
Impact factor of journals ≥3.4 vs. <3.4_[Ref]_	2.34 (0.96, 5.73)	0.06	2.77 (1.03, 7.40)	**0.04**
Published in general vs. special journal_[Ref]_	0.62 (0.2, 1.96)	0.42	0.42 (0.11, 1.61)	0.21
Funding vs. no_[Ref]_	0.71 (0.31, 1.61)	0.41	0.77 (0.29, 2.03)	0.59
**3c. Conclusion**				
English vs. others^a^_[Ref]_	1.51 (0.32, 7.09)	0.60	2.78 (0.45, 17.35)	0.27
Publication year >2013 vs. ≤ 2013_[Ref]_	0.77 (0.36, 1.66)	0.50	0.90 (0.37, 2.19)	0.82
Impact factor of journals ≥3.4 vs. <3.4_[Ref]_	2.77 (1.17, 6.58)	0.02	3.21 (1.29, 8.00)	**0.01**
Published in general vs. special journal_[Ref]_	1.76 (0.55, 5.66)	0.34	1.45 (0.41, 5.22)	0.57
Funding vs. no_[Ref]_	1.39 (0.64, 3.01)	0.40	1.82 (0.73, 4.52)	0.20
**4. Brief background summary**				
English vs. others^a^_[Ref]_	0.32 (0.67, 1.56)	0.16	0.43 (0.06, 3.20)	0.41
Publication year >2013 vs. ≤ 2013_[Ref]_	0.68 (0.26, 1.75)	0.42	1.04 (0.32, 3.39)	0.95
Impact factor of journals ≥3.4 vs. <3.4_[Ref]_	5.12 (1.34, 19.52)	0.02	6.23 (1.50, 25.71)	**0.01**
Published in general vs. special journal_[Ref]_	2.42 (0.72, 8.14)	0.15	3.15 (0.69, 14.30)	0.14
Funding vs. no_[Ref]_	1.28 (0.49, 3.32)	0.61	2.61 (0.74, 9.20)	0.14
**5a. Demographic information**				
English vs. others^a^_[Ref]_	68743610.33 (0.00, 0.00)	1.00	90494082.37 (0.00, 0.00)	1.00
Publication year >2013 vs. ≤ 2013_[Ref]_	0.34 (0.03, 3.38)	0.36	0.36 (0.03, 3.75)	0.39
Impact factor of journals ≥3.4 vs. <3.4_[Ref]_	0.91 (0.12, 6.76)	0.93	0.78 (0.10, 6.22)	0.82
Published in general vs. special journal_[Ref]_	0.00 (0.00, 0.00)	1.00	0.00 (0.00, 0.00)	1.00
Funding vs. no_[Ref]_	0.27 (0.03, 2.66)	0.26	0.24 (0.02, 2.44)	0.23
**5b. Main symptoms of the patient**				
English vs. others^a^_[Ref]_	2.04 (0.38, 11.01)	0.41	1.8 (0.3, 10.74)	0.52
Publication year >2013 vs. ≤ 2013_[Ref]_	0.69 (0.32, 1.51)	0.36	0.66 (0.28, 1.55)	0.34
Impact factor of journals ≥3.4 vs. <3.4_[Ref]_	1.12 (0.48, 2.6)	0.79	1.13 (0.47, 2.69)	0.78
Published in general vs. special journal_[Ref]_	1.86 (0.6, 5.8)	0.29	1.38 (0.4, 4.76)	0.61
Funding vs. no_[Ref]_	1.37 (0.63, 2.99)	0.42	1.29 (0.54, 3.08)	0.56
**5c. Medical, family, and psychosocial history**				
English vs. others^a^_[Ref]_	2.75 (0.58, 13.03)	0.20	1.90 (0.36, 10.15)	0.45
Publication year >2013 vs. ≤ 2013_[Ref]_	0.92 (0.41, 2.05)	0.83	1.05 (0.43, 2.55)	0.92
Impact factor of journals ≥3.4 vs. <3.4_[Ref]_	0.85 (0.35, 2.05)	0.72	0.83 (0.34, 2.05)	0.69
Published in general vs. special journal_[Ref]_	1.36 (0.39, 4.67)	0.63	1.08 (0.29, 4.01)	0.91
Funding vs. no_[Ref]_	0.93 (0.41, 2.08)	0.85	0.72 (0.29, 1.78)	0.48
**6. Physical examination findings**				
English vs. others^a^_[Ref]_	0.68 (0.07, 6.25)	0.73	0.75 (0.08, 7.36)	0.81
Publication year >2013 vs. ≤ 2013_[Ref]_	0.57 (0.16, 2.08)	0.40	0.62 (0.16, 2.31)	0.47
Impact factor of journals ≥3.4 vs. <3.4_[Ref]_	1.11 (0.31, 3.95)	0.87	0.97 (0.26, 3.6)	0.97
Published in general vs. special journal_[Ref]_	1.52 (0.29, 7.89)	0.62	1.46 (0.27, 7.87)	0.66
Funding vs. no_[Ref]_	0.67 (0.19, 2.36)	0.54	0.71 (0.19, 2.58)	0.60
**7. Important dates and times**				
English vs. others^a^_[Ref]_	0.29 (0.05, 1.56)	0.15	0.15 (0.02, 1.38)	0.09
Publication year >2013 vs. ≤ 2013_[Ref]_	0.81 (0.37, 1.76)	0.60	0.94 (0.39, 2.24)	0.88
Impact factor of journals ≥3.4 vs. <3.4_[Ref]_	1.22 (0.53, 2.84)	0.64	1.13 (0.47, 2.72)	0.78
Published in general vs. special journal_[Ref]_	1.33 (0.43, 4.11)	0.62	1.82 (0.51, 6.48)	0.36
Funding vs. no_[Ref]_	0.86 (0.40, 1.86)	0.70	1.01 (0.42, 2.44)	0.98
**8a. Diagnostic methods**				
English vs. others^a^_[Ref]_	105357054.97 (0.00, 0.00)	1.00	139445195.91 (0.00, 0.00)	1.00
Publication year >2013 vs. ≤ 2013_[Ref]_	0.20 (0.02, 1.74)	0.14	0.22 (0.02, 2.04)	0.18
Impact factor of journals ≥3.4 vs. <3.4_[Ref]_	1.9 (0.33, 10.98)	0.47	1.71 (0.28, 10.35)	0.56
Published in general vs. special journal_[Ref]_	0.00 (0.00, 0.00)	1.00	0.00 (0.00, 0.00)	1.00
Funding vs. no_[Ref]_	0.83 (0.16, 4.33)	0.83	0.79 (0.14, 4.35)	0.78
**8b. Diagnostic challenges**				
English vs. others^a^_[Ref]_	0.00 (0.00, 0.00)	1.00	0.00 (0.00, 0.00)	1.00
Publication year >2013 vs. ≤ 2013_[Ref]_	0.00 (0.00, 0.00)	1.00	0.00 (0.00, 0.00)	1.00
Impact factor of journals ≥3.4 vs. <3.4_[Ref]_	39401825.42 (0.00, 0.00)	1.00	14274663.93 (0.00, 0.00)	1.00
Published in general vs. special journal_[Ref]_	55073007.57 (0.00, 0.00)	1.00	6861059.94 (0.00, 0.00)	1.00
Funding vs. no_[Ref]_	0.00 (0.00, 0.00)	1.00	0.00 (0.00, 0.00)	1.00
**8c. Diagnostic reasoning**				
English vs. others^a^_[Ref]_	1.38 (0.25, 7.62)	0.71	2.04 (0.31, 13.26)	0.45
Publication year >2013 vs. ≤ 2013_[Ref]_	0.60 (0.24, 1.51)	0.28	0.59 (0.21, 1.64)	0.31
Impact factor of journals ≥3.4 vs. <3.4_[Ref]_	2.13 (0.78, 5.81)	0.14	2.10 (0.73, 6.03)	0.17
Published in general vs. special journal_[Ref]_	4.4 (0.54, 35.48)	0.16	3.95 (0.47, 33.17)	0.21
Funding vs. no_[Ref]_	1.01 (0.40, 2.51)	0.99	1.27 (0.45, 3.58)	0.65
**9. Therapeutic intervention**^**b**^				
English vs. others^a^_[Ref]_	0.81 (0.15, 4.45)	0.81	1.30 (0.14, 12.21)	0.82
Publication year >2013 vs. ≤ 2013_[Ref]_	2.01 (0.81, 4.98)	0.13	1.92 (0.69, 5.38)	0.21
Impact factor of journals ≥3.4 vs. <3.4_[Ref]_	0.69 (0.25, 1.87)	0.46	0.69 (0.24, 1.96)	0.48
Published in general vs. special journal_[Ref]_	1.25 (0.36, 4.40)	0.72	1.87 (0.48, 7.24)	0.36
Funding vs. no_[Ref]_	0.65 (0.27, 1.58)	0.34	0.82 (0.29, 2.31)	0.70
**10a. Clinical course of all follow-up**				
English vs. others^a^_[Ref]_	1.56 (0.28, 8.64)	0.61	2.01 (0.33, 12.17)	0.45
Publication year >2013 vs. ≤ 2013_[Ref]_	0.93 (0.36, 2.38)	0.88	0.71 (0.25, 2.01)	0.52
Impact factor of journals ≥3.4 vs. <3.4_[Ref]_	0.98 (0.36, 2.71)	0.97	1.04 (0.36, 2.98)	0.94
Published in general vs. special journal_[Ref]_	0.62 (0.17, 2.19)	0.45	0.84 (0.20, 3.54)	0.82
Funding vs. no_[Ref]_	1.24 (0.49, 3.19)	0.65	1.35 (0.48, 3.85)	0.57
**11a. Strengths and limitations**				
English vs. others^a^_[Ref]_	2.02 (0.43, 9.51)	0.38	2.95 (0.50, 17.33)	0.23
Publication year >2013 vs. ≤ 2013_[Ref]_	2.19 (0.99, 4.85)	0.05	1.61 (0.67, 3.88)	0.29
Impact factor of journals ≥3.4 vs. <3.4_[Ref]_	0.80 (0.34, 1.88)	0.61	0.84 (0.35, 2.05)	0.71
Published in general vs. special journal_[Ref]_	1.29 (0.40, 4.16)	0.67	1.46 (0.39, 5.50)	0.57
Funding vs. no_[Ref]_	0.77 (0.35, 1.68)	0.51	0.85 (0.35, 2.05)	0.71
**11b. Relevant medical literature**				
English vs. others^a^_[Ref]_	291953284.85 (0.00, 0.00)	1.00	260614308.97 (0.00, 0.00)	1.00
Publication year >2013 vs. ≤ 2013_[Ref]_	1.25 (0.42, 3.74)	0.69	1.80 (0.51, 6.35)	0.36
Impact factor of journals ≥3.4 vs. <3.4_[Ref]_	1.33 (0.39, 4.56)	0.65	1.56 (0.44, 5.57)	0.49
Published in general vs. special journal_[Ref]_	0.42 (0.05, 3.50)	0.42	0.52 (0.06, 4.71)	0.56
Funding vs. no_[Ref]_	1.83 (0.58, 5.78)	0.30	1.25 (0.35, 4.45)	0.73
**11c. Rationale for conclusions**				
English vs. others^a^_[Ref]_	315214603.48 (0.00, 0.00)	1.00	332932590.98 (0.00, 0.00)	1.00
Publication year >2013 vs. ≤ 2013_[Ref]_	0.42 (0.14, 1.32)	0.14	0.57 (0.17, 1.95)	0.37
Impact factor of journals ≥3.4 vs. <3.4_[Ref]_	1.26 (0.40, 4.00)	0.69	1.4 (0.42, 4.65)	0.58
Published in general vs. special journal_[Ref]_	0.39 (0.05, 3.21)	0.38	0.56 (0.06, 4.92)	0.60
Funding vs. no_[Ref]_	2.93 (0.88, 9.79)	0.08	2.52 (0.7, 9.08)	0.16
**11d. Main ‘take-away’ lessons**				
English vs. others^a^_[Ref]_	3.33 (0.39, 28.82)	0.27	3.47 (0.37, 32.07)	0.27
Publication year >2013 vs. ≤ 2013_[Ref]_	0.46 (0.20, 1.06)	0.07	0.53 (0.21, 1.30)	0.17
Impact factor of journals ≥3.4 vs. <3.4_[Ref]_	1.06 (0.44, 2.52)	0.90	0.99 (0.40, 2.45)	0.99
Published in general vs. special journal_[Ref]_	1.08 (0.33, 3.49)	0.90	1.34 (0.37, 4.80)	0.65
Funding vs. no_[Ref]_	0.77 (0.34, 1.73)	0.52	0.81 (0.33, 1.99)	0.65
**13. Informed consent**				
English vs. others^a^_[Ref]_	1.64 (0.35, 7.70)	0.53	1.13 (0.18, 7.10)	0.90
Publication year >2013 vs. ≤ 2013_[Ref]_	0.77 (0.36, 1.67)	0.51	0.74 (0.29, 1.86)	0.52
Impact factor of journals ≥3.4 vs. <3.4_[Ref]_	1.43 (0.62, 3.31)	0.40	1.71 (0.66, 4.38)	0.27
Published in general vs. special journal_[Ref]_	3.59 (0.94, 13.71)	0.06	7.53 (1.43, 39.76)	**0.02**
Funding vs. no_[Ref]_	3.05 (1.37, 6.78)	0.01	3.78 (1.45, 9.85)	**0.01**

Item 8d and item 12 were not include because the adherence rate was 0%

*Ref* Reference level

^&^From separate logistic regression models adjusting for other factors

^a^Others: including Chinese, Polish and Croatian

^b^The revised items by merging sub-items

## Discussion

The present study collected case reports on PNDM over the past half century. To the best of our knowledge, this is the first epidemiological study to systematically assess the extent to which case reports comply with reporting guidelines in this specific field. A total of 105 case reports for PNDM were identified. Across these case reports, this study found that the critical details regarding prognostic characteristics, patient perspectives, diagnostic challenges, follow-up visits, diagnostic reasoning, title and case presentation were often omitted. The apparent low adherence rate was primarily due to poor reporting; however, the non-mandatory requirement (patient perspective or prognostic characteristics) of the items may also affect the assessment [14]. The failure to report diagnostic information was probably due to the lack and disarray of diagnostic criteria in the area of rare diseases [5]. The under-reporting of follow-up visits could be partly because this information was not available, as the patient did not revisit the physician or died because of progressive disease [16].

Conversely, this study found that the items related to therapeutic intervention were better reported (more than 70% of case studies complied completely), such as the type, administration and changes in intervention. This finding was consistent with studies addressing the reporting quality using CARE guidelines in high-impact journals (AR=79.9%) [17], coronavirus disease (AR=84.0%) [18] and dental trauma field (AR=98.0%) [19]. A study conducted in emergency medicine used self-made 11 items scale by referring to clinical epidemiology textbooks, guidelines for critical appraisal studies, and the Users’ Guides to Evidence-Based Medicine also found similar result (AR=79.9%) [12]. Although the evaluation tools are different, these studies reflected the attentions of clinical intervention by authors, editors, and peer reviewers.

The inconsistent and suboptimal reporting across items implies that certain items may have been treated differently, as to their importance [20]. Retaining more clinically significant content and removing details about the methodology was often suggested by the editor, as journals usually pay more attention to the clinical value of research [21]. Given that some PNDM case reports were published as letters that may have strict word limitations, the deletion of “non-sense” information is even more common [12]. We would argue that while journal space is valuable, editors must balance the need to be concise with the importance of adequate case descriptions.

Both our linear and logistic regression analyses identified that the PNDM case reports published in higher impact factor journals were statistically associated with a higher reporting quality. This was consistent with the research published in 2018 and 2020 [17, 22]. Even though the use of journal impact factor as surrogate metric to measure journal quality is controversial [23], but it’s worth to mention that the overall completeness in reporting was high for CARE endorsing journals, such as the BMJ Case Reports and JAMA [17].

### Strengths and limitations

This study has several strengths. We innovatively assessed the quality of the PNDM case reports using the widely accepted CARE checklist. Second, a comprehensive search, explicit eligibility criteria, rigorous methods for screening studies and data collection ensured transparency and reproducibility of judgments. Third, the use of two independent reviewers for the preselection of case reports, assessment quality and data extraction was of great help in avoiding errors and subjective judgments.

This study has some limitations. First, the results were confined to PNDM case reports, which constituted a small fraction of case reports. Second, we scored reporting quality and added a category “Partially yes” to each item that may skew the results. Third, we did not include any grey literature and the reporting quality of these case reports was unknown. We expect such a report to be rare. Fourth, the non-mandatory requirement of some items may underestimate the results of the reporting quality.

## Conclusion

Reporting of PNDM case reports is generally suboptimal. Substantial effort is needed to improve reporting, especially the reporting of case presentation, diagnostic assessment, follow-up, and outcomes. A larger word count may be beneficial for better reporting. To improve reporting quality, journals should encourage strict adherence to the CARE guidelines.

### Supplementary Information

**Supplementary Material 1.**

**Supplementary Material 2.**

## Data Availability

All data generated or analysed during this study are included in this published article and its supplementary information files.

## Abbreviations

AR: Adherence rate
CI: Confidence interval
CARE: CAse REport
IQR: Interquartile range
NDM: Neonatal diabetes mellitus
OR: Odds rate
PNDM: Permanent neonatal diabetes mellitus
