# Supplementary material for: The quality of reporting in case reports of permanent neonatal diabetes mellitus: a cross-sectional study
Source: BMC Med Res Methodol. 2024 May 20;24:117. doi: 10.1186/s12874-024-02226-1 (PMC11103994; doi:10.1186/s12874-024-02226-1)
Supplement: Supplementary file 1 — Supplementary Material 1. [file 12874_2024_2226_MOESM1_ESM.docx]

**Pubmed**

1.(Diabetes Mellitus, Permanent Neonatal)[mh]

2. Permanent[tiab] AND Neonatal[tiab] AND Diabetes[tiab]

3.case reports[pt]

4.case[tw] AND (report* OR stud*)[tw]

5. 1 or 2

6.3 or 4

7. 5and 6

**Embase**

1.'permanent neonatal diabetes mellitus'/exp

2.permanent:ti,ab,kw AND neonatal:ti,ab,kw AND diabetes:ti,ab,kw

3.report*:ti,ab,kw OR stud*:ti,ab,kw

4.case:ti,ab,kw

5.'case report'/exp

6. 1 or 2

7. 3 or 4 or 5

8. 6 and 7

**CINAHL**

1.(TI (Permanent Neonatal Diabetes Mellitus) OR

2.(AB (Permanent Neonatal Diabetes Mellitus)) OR

3.(TI (Permanent) AND TI (Neonatal) TI (Diabetes)) OR

4.AB (Permanent) AND AB (Neonatal) AB (Diabetes) )

5.(MW (case studies) OR (TI (case) AND TI (stud*) OR TI (report*)) OR (AB (case) AND AB (stud*) OR AB (report*)) )

6. 1 or 2 or 3 or 4

7. 5 and 6

**Scopus**

1.TITLE-ABS-KEY ( permanent AND neonatal AND diabetes )

2.TITLE-ABS-KEY ( case AND (report* OR stud*))

3.1 and 2

**Web of Science**

(TS=(Permanent) AND TS=(Neonatal) AND TS=(Diabetes)) AND TS=(case) AND (TS=(report*) OR TS=(stud*)))

**Medrxiv**

1.(permanent AND neonatal AND diabetes)title, abstract, and full text

2.(case AND (report* OR stud*))

3.1 and 2

**SinoMed**

(neonatal diabetes mellitus (Title, Abstract) or permanent (Title, Abstract)) and case report (Title, Abstract)

**CNKI**

1.neonatal diabetes mellitus (Title, Keyword，Abstract) or permanent (Title, Keyword，Abstract)

2.case report (Title, Keyword, Abstract)

3. 1 and 2

**Wanfang**

(neonatal diabetes mellitus (Mesh, Keyword) or permanent (Mesh, Keyword)) and case report (Mesh, Keyword)

**VIP**

(neonatal diabetes mellitus (Title, Keyword) or permanent (Title，Keyword)) and case report (Title, Keyword)
