# Supplementary material for: The quality of reporting in case reports of permanent neonatal diabetes mellitus: a cross-sectional study
Source: BMC Med Res Methodol. 2024 May 20;24:117. doi: 10.1186/s12874-024-02226-1 (PMC11103994; doi:10.1186/s12874-024-02226-1)
Supplement: Supplementary file 2 — Supplementary Material 2. [file 12874_2024_2226_MOESM2_ESM.docx]

The revised 23 CARE guidelines checklist

| **Items** | **Item**  **number** | **Brief description** |
| --- | --- | --- |
| Title | 1 | The words ‘case report’ (or ‘case study’) should appear in the title along with phenomenon of greatest interest (eg, symptom, diagnosis, test, intervention) |
| Keywords | 2 | The key elements of this case in 2–5 words |
| Abstract | 3a | Introduction—What does this case add? |
|  | 3b**^#^** | the main symptoms of the patient, main clinical findings, the main diagnoses and interventions and the main outcomes |
|  | 3c | Conclusion—What were the main ‘take-away’ lessons from this case? |
| Introduction | 4 | Brief background summary of this case referencing the relevant medical literature |
| Patient information | 5a | Demographic information (eg, age, gender, ethnicity, occupation) |
|  | 5b | Main symptoms of the patient (his or her chief symptoms) |
|  | 5c | Medical, family, and psychosocial history—including diet, lifestyle, and genetic information whenever possible, and details about relevant comorbidities including past interventions and their outcomes |
| Clinical findings | 6 | Describe the relevant physical examination (PE) findings |
| Timeline | 7 | Depict important dates and times in this case (table or figure) |
| Diagnostic assessment | 8a | Diagnostic methods (eg, PE, laboratory testing, imaging, questionnaires) |
|  | 8b | Diagnostic challenges (eg, financial, language/cultural) |
|  | 8c | Diagnostic reasoning including other diagnoses considered |
|  | 8d | Prognostic characteristics (eg, staging) where applicable |
| Therapeutic intervention | 9**^#^** | Types of intervention (eg, pharmacologic, surgical, preventive, self-care)  – Administration of intervention (eg, dosage, strength, duration)  – Changes in intervention (with rationale) |
| Follow-up and outcomes | 10**^#^** | Summarise the clinical course of all follow-up visits including  – Clinician and patient-assessed outcomes;  –Important follow-up test results (positive or negative);  –Intervention adherence and tolerability (and how this was assessed);  –Adverse and unanticipated events |
| Discussion | 11a | The strengths and limitations of the management of this case |
|  | 11b | The relevant medical literature |
|  | 11c | The rationale for conclusions (including assessments of cause and effect) |
|  | 11d | The main ‘take-away’ lessons of this case report |
| Patient perspective | 12 | The patient should share his or her perspective or experience whenever possible |
| Informed consent | 13 | Did the patient give informed consent? Please provide if requested |
| Note: **^#^**: The revised items by merging sub-items | | |
